# Tips and tricks for endoscopic negative pressure therapy

**DOI:** 10.1007/s00104-018-0725-z

**Published:** 2018-10-02

**Authors:** G. Loske, C. T. Müller

**Affiliations:** Klinik für Allgemein‑, Viszeral‑, Thorax und Gefäßchirurgie, Katholisches Marienkrankenhaus Hamburg gGmbH, Alfredstr. 9, 22087 Hamburg, Germany

**Keywords:** Endoscopic vacuum therapy, Leakage, Drainage, Overtube, Esophagus, Endoskopische Vakuumtherapie, Leckage, Drainage, Overtube, Ösophagus

## Abstract

Endoscopic negative-pressure therapy (ENPT) is becoming a valuable tool in surgical complication management of transmural intestinal defects and wounds in the upper and lower gastrointestinal tract. Innovative materials for drains have been developed, endoscopic techniques adapted, and new indications for ENPT have been found. Based on our broad clinical experience, numerous tips and tricks are described, which contribute to the safety of dealing with the new therapy. The aim of this work is to present these methods. The focus is on describing the treatment in the esophagus.

## Zusammenfassung

Die endoskopische Unterdrucktherapie (EUT) entwickelt sich zu einem wertvollen Werkzeug im chirurgischen Komplikationsmanagement von transmuralen intestinalen Defekten und Wunden im oberen und unteren Gastrointestinaltrakt. Innovative Drainagematerialien wurden entwickelt, endoskopische Techniken adaptiert und neue Indikationen zur EUT gefunden. Aus der klinischen Anwendung lassen sich zahlreiche Tipps und Tricks beschreiben, die zur Sicherheit im Umgang mit der neuen Therapie beitragen. Ziel dieser Arbeit ist es, diese Methoden darzustellen. Der Schwerpunkt der Darstellungen liegt auf der Anwendungsbeschreibung im Ösophagus.

## Infobox 1 Notes on the initial endoscopic examination of gastrointestinal defects

*Initial endoscopic examination*

- CO_2_ insufflation
- Short examination time
- No debridement
- No or little irrigation
- Small openings: thin endoscope
- Goal: creation of a compartment

Endoscopic negative pressure therapy (ENPT; also known as endoscopic vacuum therapy, endovac therapy, E‑Vac therapy) is developing into an innovative surgical/endoscopic option for treating intestinal leaks throughout the entire gastrointestinal tract. After this method was first used to treat rectal anastomosis insufficiencies [1], it attracted the attention of surgeons and gastroenterologists as a way of managing postoperative complications of intrathoracic esophagus anastomoses and of treating esophageal perforations [2]. Since then, as reported in several recently published retrospective studies, it has been used on more than 210 patients with esophageal defects with a recovery rate of approximately 90% [3]. Innovative drainage materials have been developed and endoscopic techniques adapted accordingly. This has allowed for other uses of ENPT and resulted in new indications.

The information in this article was presented at the 2017 annual congress of the German Society for Surgery in the session by the German Society for General and Visceral Surgery (DGAV)/Surgical Working Group for Endoscopy and Sonography (CAES) entitled “Upper GI Tract Insufficiency: Changes in Diagnosis and Treatment.” To help promote ENPT and ensure its safe performance, we would like to share tips and tricks, including new methods and materials, with interested colleagues, drawing on our experience in numerous clinical cases. The focus of the information we present here is the use of this technique in the esophagus.

## Endoscopy of gastrointestinal leaks

General preliminary remarks about endoscopy when intestinal leakage is present or suspected are listed in Infobox 1.

We perform all endoscopies with CO_2_ insufflation to reduce the potential risk of an air embolism and to avoid the dissemination of difficult-to-absorb air through a defect and the wound. We typically use standard gastroscopes with diameters of up to 10 mm.

We always try to keep the examination time to a minimum during the initial examination, we do not perform debridement, and we only irrigate inner wound cavities slightly. The rationale for this is that early defects and infected wounds are more permeable to the gases and fluids used for the examination. The infection could spread as a result of the examination. During the initial treatment interval, our primary treatment goal is to create a local wound compartment that can then undergo further local therapy.

We do not perform debridement of necrotic tissue, if required at all, until later in the treatment course. Necrotic tissue is demarcated during ENPT, allowing for gentle removal and avoiding further trauma.

**Fig. 1:**
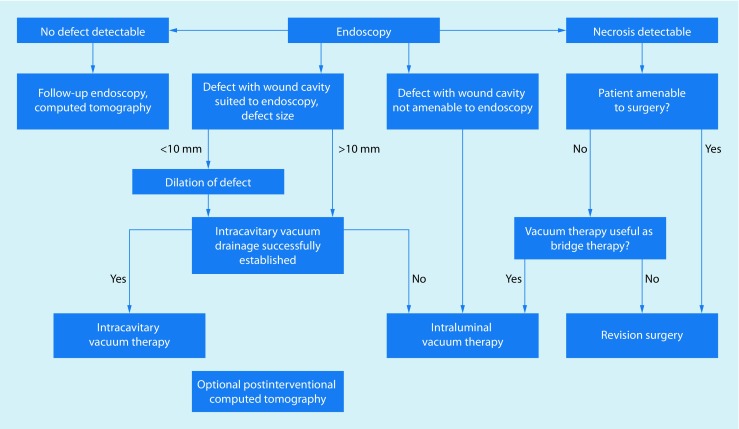
Treatment algorithm for endoscopic negative pressure therapy in the esophagus (taken from [4], this content is not part of the Open Access licence)

If minor transmural defects are present, we initially explore them using small-caliber nasal endoscopes (approximately 5 mm in diameter). Only after this has been done do we decide whether dilatation of the defect is necessary to perform intracavitary ENPT. When performing this procedure, we follow a treatment algorithm (Fig. 1; [4]), which has also been adopted by other users [5, 6].

## Local requirements for performing ENPT of the esophagus

Endoscopic negative pressure therapy can be used for the treatment of transmural defects irrespective of location (including the gastroesophageal junction and high cervical esophagus), size, or infection status.

Two basic requirements must be met: intact or at least compensated perfusion and the presence of a closed compartment, allowing for the build-up of negative pressure. Exclusion criteria for ENPT as monotherapy are therefore longitudinal necrosis of the esophagus after abdominothoracic esophageal resection and a connection to the tracheobronchial system.

A further important criterion is the collapse of the wound cavity under suction. Occasionally, the compartment forms only when tissue attaches to the foam. The wound margins adjust or tissue attaches to the defect from outside.

## Intraluminal and intracavitary versions of ENPT and endoscopic techniques

**Fig. 2:**
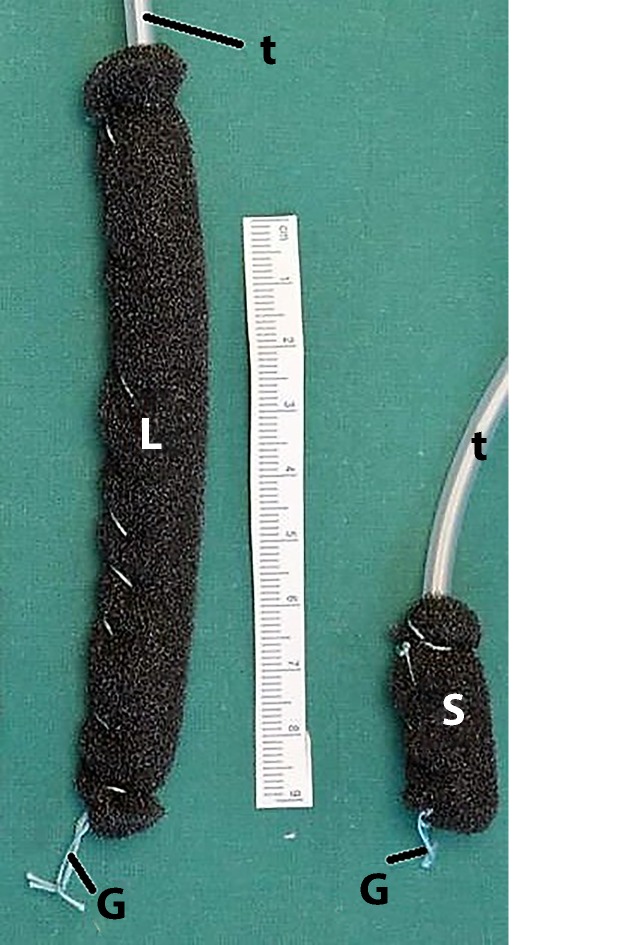
Open-pore polyurethane foam drains; a long (*L*) or short (*S*) piece of polyurethane foam is affixed around the distal end of the drain tube (*t*); an endoscopic grasper can be used to hold the thread loop (*G*)

Open-pore drains are required for ENPT. They consist of a drain tube with an open-pore polyurethane foam (*o*pen-pore *p*olyurethane foam *d*rain [OPD]) affixed around the distal end. We use short and long foam pieces (Fig. 2). The OPD is placed in the wound area endoscopically and defined negative pressure is applied over several days using an electronic pump. The drain is changed at intervals of 3–5 days and the course of wound healing is endoscopically evaluated. Depending on wound status, the endoscopic treatment can be supplemented by performing necrosectomy, debridement, or irrigation. We irrigate with between 100 and 500 ml of fluid, also using the Endowasher. The fluid is always completely suctioned endoscopically and bacteriology tests can be performed. To date, we have not used any antiseptic additives to the solution.

All conventional endoscopic techniques are used to place the OPD. We use the push, pull, pull-through, and piggyback techniques most frequently, but insertion using guide wires, the Seldinger technique, and surgical rendezvous are also possible (Table 1).

**Table 1:** Endoscopic techniques in endoscopic negative pressure therapy

Endoscopic technique	Description of the endoscopic maneuver
Push	The open-cell drainage element (OD) is advanced to the desired location with an endoscopic grasper (e. g., polyp grasper, forceps) or along an overtube with a pusher or the endoscope
Pull	The OD is placed by pulling back on the drain tube under endoscopic control (e. g., via an anastomosis)
Pull-through	The OD is pulled to the desired location along a preformed channel (e. g., an enterocutaneous fistula)
Piggyback	A thread loop held with an endoscopic grasper is affixed to the distal end of the OD. The OD (riding on the back of the endoscope) is introduced together with the endoscope
Rendezvous	Surgical placement of the OD
Seldinger technique	The OD can be advanced to the desired location along a guidewire that has been endoscopically inserted beforehand
Guidewire	A guidewire is inserted as a guide into the drain tube

**Fig. 3:**
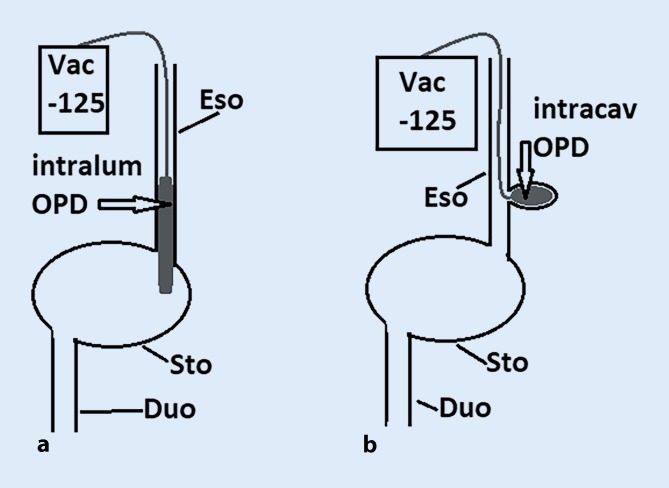
Diagram of the treatment versions (**a** intraluminal negative pressure therapy [*intralum OPD*], **b** intracavitary negative pressure therapy [*intracav OPD*]) in the esophagus (*Eso*) with open-pore polyurethane foam drains (*OPD*); stomach (*Sto*); duodenum (*Duo*); vacuum pump with −125 mm Hg negative pressure (*Vac -125*)

We differentiate between two ENPT versions: intraluminal and intracavitary ([7]; Fig. 3). The two versions can also be used together and combined.

In intracavitary ENPT, the OPD foam piece is introduced through the wall defect into an extraluminal cavity. In the esophagus, we usually use short foam pieces with a diameter of around 1.5 cm and a length of 2–4 cm. A short foam piece increases the endoscope’s maneuverability and makes it easier to put in place. An important criterion is the collapsing of the wound cavity under suction. The aim is to empty the cavity as completely as possible with the applied negative pressure. Suctioning during drainage causes closure of the defect around the drain tube. The foam pieces can also be placed in such a way that the oral part projects luminally out over the defect and sits like a cork in the opening. The wall defect adheres via suction to the foam and is sealed.

We prefer to use the intracavitary version of ENPT for the treatment of extraluminal wound cavities that we can reach with the endoscope along the transmural defect. For minor defects we use small-caliber endoscopes for the initial examination in order to assess the extraluminal wound status. If we find an infected extraluminal cavity requiring drainage, we also dilate the defect so that we can insert an OPD. If we find only a small cavity with no infection, we use a novel small-lumen *o*pen-pore *f*ilm *d*rain (OFD, see next section) or also the intraluminal version of ENPT with an OPD.

In intraluminal ENPT, the negative pressure is applied directly in the intestinal lumen. In the esophagus, we use long foam pieces with a diameter of around 1.5 cm and a length of up to 12 cm. The open-pore foam should cover the defect zone or anastomosis completely. Ideally, the foam is positioned so that the defect zone is located in the center of the foam. The foam should bridge the defect zone along the oral–aboral axis. After negative pressure has been applied, the intestinal lumen will collapse with and around the foam. This can result in a complete therapeutic closure of the esophagus, for example.

The drain is changed endoscopically every 3–5 days. The condition of the wound interior is inspected and wound healing progress is assessed endoscopically. The decision is then made to either continue or end the treatment. If the wound status so requires or the function of the drain needs checking for other reasons, a change of drain may be indicated also at shorter intervals.

## Open-pore polyurethane foams, erosive suction patterns, and electronically controlled negative pressure

We have been using open-pore polyurethane foams, which are known from the treatment of external wounds, also for negative pressure therapy from the very beginning (V.A.C.® Granufoam^TM^, KCI, San Antonio, TX, USA; Suprasorb® CNP Wound Foam, Lohmann & Rauscher GmbH & Co. KG, Neuwied, Germany). It is only in recent years that an approved medical device (EsoSPONGE®, B. Braun Melsungen AG, Melsungen, Germany) has been commercially available for the esophagus. This is an OPD with a short open-pore foam piece that is introduced with a pusher along an overtube that is approximately 60 cm in length. The application technique is comparable to that of the EndoSPONGE® drain (B. Braun Melsungen AG, Melsungen, Germany), which is currently the only medical device available for use in the rectum. The user has to adjust the size of the approved medical devices over the course of the treatment, just as they do for self-made devices, using scissors to shorten or narrow the foam. ENPT requires the user to actively shape the components and be creative when handling them each time they are used.

All polyurethane foams are characterized by densely packed pore openings. Depending on the size of the foam surface, the pore size, and the consistency of the wound surface, they can be suctioned very firmly to the wound bed and accordingly adhere tightly. This phenomenon is known from using negative pressure therapy on the body surface. Under suction, a granulating wound bed “gets stuck” in the numerous pores. In contrast to reports in the literature [8], we have never observed genuine growth of tissue into the sponge when changed at intervals of 3–5 days. However, the sponge head may adhere to the wound bed so tightly that owing to high-tensile forces during removal the foam may tear off from the drain or the drain tube itself may even tear.

**Fig. 4:**
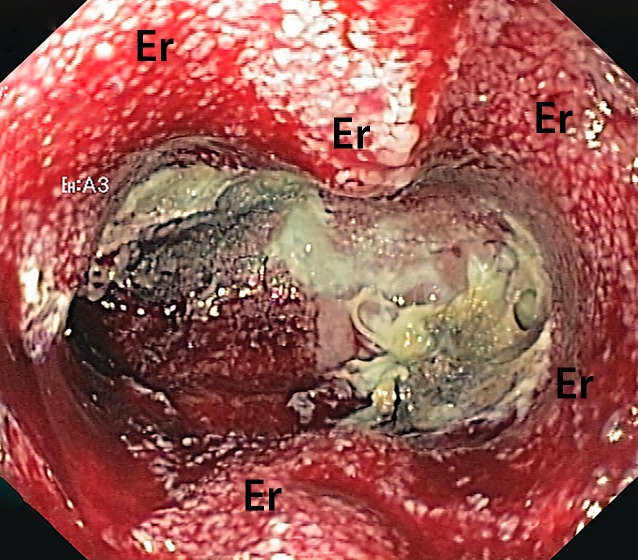
Wound cavity following removal of open-pore polyurethane foam that had fixed itself firmly to the wound bed under suction. Typical erosions (*Er*) can be seen, no necrotic tissue has yet been shed in the depth of the wound

In ENPT, adherence of the foam to the wound bed is an important sign that the therapy is working well. Typically, one can see erosive patterns (Fig. 4) on the wound bed after removal. If the sponge adheres so tightly that considerable force would be required to remove it, we turn off the suction for 24 h and wait until the next day before once again attempting removal. Removal is then usually relatively easy because the suctioned tissue detaches from the foam. In addition, we also try to get between the sponge and tissue with the blunt tip of the endoscope and an endoscopic grasping device to separate it with careful lateral movements. To date, we have always been able to completely remove the foreign material endoscopically. We do not rinse the drain prior to removal. There have been rare reports of bleeding complications due to erosions when using OPDs for extraluminal intracavitary ENPT in esophageal defects [6, 9].

To date, no negative pressure pumps have been developed for ENPT and approved as medical devices. We use electronic pumps solely (V.A.C. Freedom® and Activac®, KCI, San Antonio, TX, USA). For us, it is very important that negative pressure is generated and adjusted as rapidly as possible, that suction is continuous, and that the alarms function properly. We consider pumps that slowly build up suction over several seconds, drainage bottles, wall suctioning, and thoracic drainage pumps as unsuitable for use in the esophagus. Our standard negative pressure is −125 mm Hg for both the intraluminal and intracavitary treatment versions.

## Three-prong polyp grasper, forceps, nasolabial fixation

**Fig. 5:**
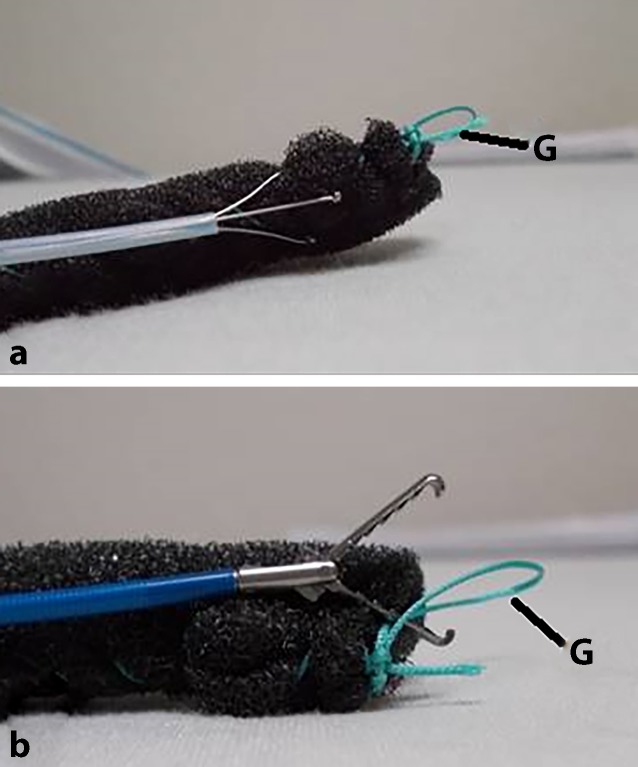
Different options for holding the foam piece with an endoscopic grasper; a thread loop (*G*) at the distal end makes placement easier; **a** with a three-prong grasper, **b** with forceps

To place the open-pore sponge, we grasp it directly on the distal end with a three-prong polyp grasper. Alternatively, endoscopic forceps can also be used. A thread loop at the distal end of the drain can make placement easier (Fig. 5).

After oronasal redirection of the drain tube, we recommend fixation with a suture to the nasolabial fold (no fixation to the nasal septum or the nasal wing). In cooperative patients, a nasal bridle or plaster may also be used for fixation. For the intraluminal version, fixation prevents distal propulsion-induced dislocation or accidental removal by pulling on the drain tube.

## Overtube

**Fig. 6:**
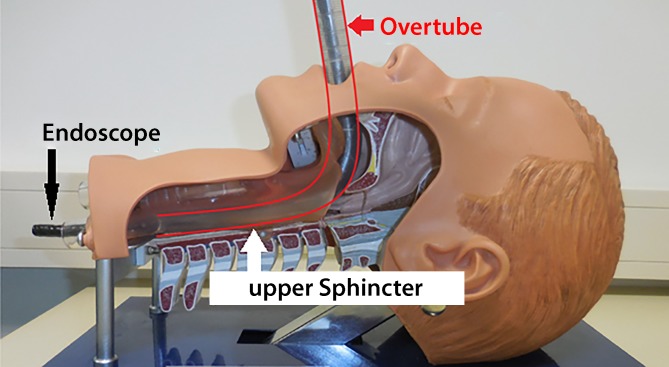
Use of a short overtube to splint the pharynx and oral esophagus makes the procedure safer and the introduction of open-pore foam drains easier. The anatomical conditions can be seen in the model

During the placement maneuver, the endoscope and drain must negotiate the oropharyngeal curve and the upper esophageal sphincter. The patients are frequently intubated, which makes it more difficult to see in the mouth and throat. To ensure the throat, pharynx, and oral esophagus are bypassed safely during the insertion maneuver, we use an approximately 30-cm-long overtube (Fig. 6). It acts as an important safeguard to prevent iatrogenic perforations and makes it much easier to insert the OPD. Once the drain reaches the oral esophagus, the placement maneuver is continued in this method using endoscopic visual control.

## Intestinal feeding during intraluminal ENPT

The therapeutic closure of the esophagus during intraluminal ENPT complicates intestinal feeding, but integrating a feeding tube into the foam can circumvent this problem [10, 11] and also allows for enteral feeding. If the feeding tube is to be fixed in the drainage device, we use a triple-lumen tube (Freka®Trelumina, CH/Fr 16/9, 150 cm, Fresenius Kabi AG, Germany), wrapping polyurethane foam (Suprasorb® CNP Foam, Lohmann & Rauscher GmbH & Co. KG, Neuwied, Germany) around the gastric drain opening. The venting branch of the tube is closed, since it is not being used. Alternatively, a jejunal feeding tube can also be guided through the sponge next to the drain tube. The latter approach has the advantage that the length of the intestinal branch can be varied as desired. We introduce the jejunal feeding tube deep into the small intestine.

We begin with tea and water and observe whether transport takes place. In rare cases, food blocks the tube and is suctioned up by the pump. If this happens, feeding via the feeding tube is temporarily suspended. In rare cases, the blockage may also be caused by the feeding tube twisting on entering the stomach. This requires follow-up endoscopic examination with repositioning or reintroduction of the feeding tube.

If this type of drainage is used in patients who have undergone abdominal/thoracic esophageal resection, the distal part of the foam extends into the stomach. This distal part of the foam has then been observed to permanently drain postoperative gastric and duodenal reflux.

## Visible surgical drain in an intestinal defect, enterocutaneous fistula? Use the pull-through technique!

If there is a connection to a surgical cutaneous drain or catheter as a result of an intestinal defect, this access route can be used for a simple technique for passage drain placement, such as the pull-through technique or a rendezvous maneuver. In appropriate cases, the method described here can considerably simplify the occasionally difficult endoscopic placement maneuver. These are initial individual observations from clinical practice.

We use new types of drains where the open-pore drainage element is placed in the middle of a long tube [12–14]. The placement maneuver is similar to the thread pull-through method in percutaneous endoscopic gastrostomy. One of the tube ends is pulled cutaneously outward along the enterocutaneous connection, pulling the open-pore drainage element attached in the middle of the drain into the desired location. The drainage element can be pulled through the defect opening and placed extraluminally. It may also be pulled only partially into or before the opening. The other end of the tube is fed out nasally and suction attached. The lumen is closed at the cutaneous end. Alternatively to a tube, a thread attached to the foam end or an endoscopic grasper can also be used to pull the drain through the fistula. If a thread is used, it should be noted that the thread may cut into the tissue.

During the change maneuver, an attempt is made to reduce the diameter of the drain incrementally to a drain diameter of only 4–6 mm. We stop the ENPT when the wound has shrunk with the diameter of the drain to a stable wound channel and is coated with granulation tissue. Reducing the size of the drain diameter is accomplished by means of an innovative OFD, which will be presented in the following section.

## New open-pore materials for ENPT

**Fig. 7:**
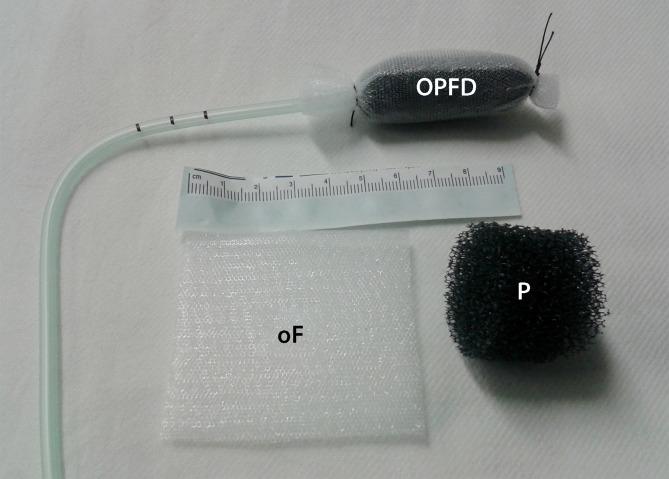
Wrapped polyurethane foam drain (*OPDF*) with open-pore film (*oF*), polyurethane foam piece (*P*)

One disadvantage of OPDs is their diameter of 1.5–3 cm, which makes their insertion into small openings difficult, if not impossible. A very thin open-pore double-layer drainage film (Suprasorb® CNP Drainage Film, Lohmann & Rauscher International GmbH & Co, Rengsdorf, Germany) has been developed for negative pressure therapy of the abdomen. It is composed of two perforated membranes separated by a cavity that does not collapse under suction. This allows for open-pore drainage of liquids along the film membranes, through them, and in the cavity between them. Negative pressure is generated over the entire surface of the drainage film. The distance between the pores in foam and film differs. In foam, the pores are densely packed and there is little distance between them. In film, the pores are arranged at a regular and close, bridge-like distance from one another. This film can be used instead of polyurethane foam and can be wrapped as an open-pore drainage element around the opening of a drain tube. This allows very small caliber open-pore drains to be created with a diameter of just a few millimeters [15], which can be inserted easily into small openings. For treatment in the upper gastrointestinal tract, the OFD can be introduced transnasally like a feeding or stomach drainage tube. A variety of new drain types can be constructed. Open-pore film and foam systems can be combined. Polyurethane foam pieces can also be wrapped with film (Fig. 7; [13, 16]).

**Fig. 8:**
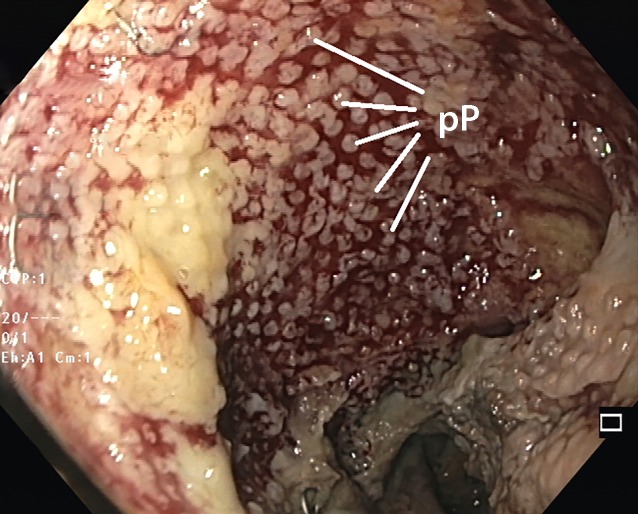
Wound cavity following removal of a film-wrapped polyurethane foam drain. Typical regular suction patterns (*pP*) can be seen

OFDs and film-wrapped foam do not adhere so firmly to the wound bed, and therefore the debridement effect is not so marked as with foam. Following removal, typical nub-like suction patterns can be found (Fig. 8). The disadvantages of OPDs caused by excessive adhesion to tissue can be overcome by wrapping them with film.

We generally begin intracavitary ENPT with OPD drains, which we always trim individually to the appropriate size and length as the treatment progresses. Recently, we have begun to make increasing use of trimmed film-wrapped OPDs for wound cleansing and reduction. We use OFD film drains when shrinking wound channels with small openings develop. Once negative pressure is applied, suction is achieved covering the entire area, secretions are drained, and suction is applied to the wound surface. In the case of a wound channel, this results in further wound conditioning.

Since in intraluminal ENPT in the esophagus good adhesion is essential to seal the defect completely, we use OPDs and not OFDs or film-wrapped OPDs. If the primary aim is to drain liquid secretions only (e. g., gastric or duodenal reflux), both OFDs and OPDs can be used. OFDs and OPDs are both effective in draining liquid secretions.

In open-pore drains, suction is maintained due to the numerous pores, even when some of the pores become blocked. Drains may also become completely blocked by viscous secretions (e. g., swallowed bronchial mucus) and lose their suction capacity. This is another reason why they require regular changing.

Open-pore drains (both OPDs and OFDs) differ from conventional passive drains, which use gravity to drain fluids along a positive pressure gradient or capillary pressure. By contrast, open-pore drains to which negative pressure is applied are active drains that also drain against gravity or capillary pressure along a negative pressure gradient in a directed manner.

With the new materials now available, other innovative treatment indications are currently in development [13–21]. Experience is still limited to a few individual reports. An example of a practical application of the OFD, which has become established during the past few months at our hospital, is the routine use of film drains for the complete prevention of gastric reflux after abdominothoracic distal esophagus resection with intrathoracic anastomosis to protect the intrathoracic anastomosis [21]. Instead of passively draining the postoperative reflux with a stomach drainage tube using gravity, we are now using active negative pressure drains in the early postoperative phase to render the stomach “dry” and so ensure that the anastomosis is not contaminated with the reflux. Our initial observations show that this has a very positive effect on the healing of the anastomosis. Further evaluation studies are required to provide adequate evidence.

## Summary of ENPT techniques

We usually initiate endoscopic negative pressure therapy using an OPD. The drains are changed regularly every 3–5 days under endoscopic inspection of the wound situation. The size of the foam drain is adapted to the wound circumstances by trimming the sponge, and the diameter is also continually reduced. With very small lumina, we switch to an OFD. With a granulating wound bed and contact to vulnerable structures, we use film-wrapped foam drains. Intracavitary and intraluminal versions of ENPT can be combined. With intraluminal ENPT, double-lumen drains with an intestinal feeding tube (with both OPDs and OFDs) allow for simultaneous intestinal feeding.

## Outlook

ENPT is an extremely effective treatment option for leaks in the gastrointestinal tract and an extremely valuable innovation in visceral surgery. Three interdependent areas are particularly important for its further development: the use of flexible endoscopy in the surgical management of complications, the availability of suitable approved medical devices, and the evaluation of the methods in scientific studies.

## Flexible endoscopy in surgery

ENPT was developed at German departments of surgery within the space of a few years. It was only in 2006 that we conducted our first negative pressure treatment in the esophagus and in 2007 that we presented it at the summer conference of the Northwest German Association of Surgeons [22]. In 2010 we reported on the first case series of ten patients [2]. Our positive results were then confirmed in several retrospective studies predominantly conducted in German departments of surgery with surgical endoscopy expertise [3]. Individual working groups within these departments have already reported on significant sample sizes of between 35 and 77 patients, demonstrating the value placed on the innovative method within this short period [4–6]. Treatment success requires expertise in both surgical wound treatment and in flexible endoscopy examination techniques. In our view, the practice of ENPT lies clearly within the remit of surgeons or at minimum requires close interdisciplinary collaboration. Determining the indication, assessing the course of wound healing, determining the end of treatment or change of procedure all require evaluation by a surgeon well versed in endoscopy or one experienced in vacuum therapy in close cooperation with the gastroenterologist performing the endoscopy. Appropriate endoscopy skills should be part of surgical training to ensure use of these treatments in the surgical management of complications and to maintain endoscopy expertise in departments of surgery. There is a need for interdisciplinary training and continuing education courses.

In 1926, Martin Kirschner [23] formulated the principles for the treatment of acute suppurative inflammation of the abdomen, stating that “the source of the infection should be eliminated as quickly and non-traumatically as possible.” The essential basic principles “involve blocking the source of the infection and managing and draining the exudate.” These principles are put into practice with ENPT.

## Materials (drains and pumps)

ENPT of the upper gastrointestinal tract has developed from clinical practice in the surgical management of complications. There is in fact a discrepancy between the single medical device first approved for use in the esophagus in Germany in 2014 (in Austria and Switzerland in 2016; EsoSPONGE®, B. Braun Melsungen AG, Melsungen, Germany) and the numerous reports of an effective, increasingly used treatment with self-made devices. Retrospective studies have been conducted with the latter, and in recent reports the further development from a purely therapeutic to preemptive use of ENPT [24] and initial thoughts on prophylactic and preventative use have been described.

A further shortcoming concerns the generation and maintenance of negative pressure, which is the second essential technical component and which to date has received scant attention. There is currently no electronic negative pressure pump available that has been specifically developed as a medical device for ENPT.

In any description of the latest technological developments and the broad and developing spectrum of the use of ENPT, it is impossible to describe the method without these modified materials and the off-label use of various components. This means that in many cases of ENPT use, no medical device safety is formally a given. Solutions must be jointly sought with partners in industry.

## Studies

The greatest expertise in ENPT is currently to be found in Germany. There is, however, a dilemma; we have and use a highly effective procedure but one for which the scientific evidence to date is based solely on retrospective case series. From a scientific point of view, therefore, the procedure should also ideally be validated in prospective, randomized studies.

Open questions, such as determining the limits of the treatment and the criteria for a change of treatment and how the treatment can complement and be combined with surgical treatment procedures [25], as well as the description of beneficial factors and limitations, can be worked on. Furthermore, the description of the physical properties of available and new materials, the simplification of methods, and the examination of specific mechanisms of action can also help ENPT be employed more effectively and in a more differentiated way. In this context, approved medical devices are also essential for comparison and standardization purposes.

## Conclusion

- Over the past few years, endoscopic negative pressure therapy has developed into a valuable tool for intracorporeal wound treatment. It enhances and supplements the management of surgical complications.
- The surgical treatment principles formulated by Martin Kirschner are still being used today and are put into practice with endoscopic negative pressure therapy. This allows for both endoscopic closure of the defect and active and targeted management of exudate.
- With ENPT, endoscopy has a particularly important role to play in surgery. The value of ENPT must be demonstrated in further studies. The development of new approved medical devices is required for drain and pump components.

## References

[1] Weidenhagen R, Gruetzner KU, Wiecken T (2008). Endoscopic vacuum-assisted closure of Anastomotic leakage following anterior resection of the rectum: a new method. Surg Endosc.

[2] Loske G, Schorsch T, Muller C (2010). Endoscopic vacuum sponge therapy for esophageal defects. Surg Endosc.

[3] Kuehn F, Loske G, Schiffmann L, Gock M, Klar E (2017). Endoscopic vacuum therapy for various defects of the upper gastrointestinal tract. Surg Endosc.

[4] Schorsch T, Müller C, Loske G (2014). Endoscopic vacuum therapy of perforations and anastomotic insufficiency of the esophagus. Chirurg.

[5] Bludau M, Fuchs HF, Herbold T, Maus MKH, Alakus H, Popp F, Leers JM, Bruns CJ, Hölscher AH, Schröder W, Chon SH (2018). Results of endoscopic vacuum-assisted closure device for treatment of upper GI leaks. Surg Endosc.

[6] Laukoetter MG, Mennigen R, Neumann PA, Dhayat S, Horst G, Palmes D, Senninger N, Vowinkel T (2017). Successful closure of defects in the upper gastrointestinal tract by endoscopic vacuum therapy (EVT): a prospective cohort study. Surg Endosc.

[7] Loske G, Schorsch T, Müller C (2011). Intraluminal and intracavitary vacuum therapy for esophageal leakage: a new endoscopic minimally invasive approach. Endoscopy.

[8] Schniewind B, Schafmayer C, Both M, Arlt A, Fritscher-Ravens A, Hampe J (2011). Ingrowth and device disintegration in an intralobar abscess cavity during endosponge therapy for esophageal anastomotic leakage. Endoscopy.

[9] Pournaras DJ, Hardwick RH, Safranek PM, Sujendran V, Bennett J, Macaulay GD, Hindmarsh A (2018). Endoluminal vacuum therapy (E-Vac): a treatment option in Oesophagogastric surgery. World J Surg.

[10] Loske G, Aumiller J, Rucktäschel F, Schorsch T (2016). Spontaneous perforation of an intramural esophageal pseudodiverticulosis treated with intraluminal endoscopic vacuum therapy using a double-lumen vacuum drainage with intestinal feeding tube. Endoscopy.

[11] So Young L, Kun Woo K, Jae-Ik L, Dong-Kyun P, Kook-Yang P, Chul-Hyun P, Kuk-Hui Son (2018). Esophageal endoscopic vacuum therapy with Enteral feeding using a Sengstaken-Blakemore tube. Korean J Thorac Cardiovasc Surg.

[12] Fischer A, Thimme R, Hopt UT, Richter-Schrag HJ (2016). Two-sided sponge (TSS) treatment: Description of a novel device and technique for endoscopic vacuum treatment (EVT) in the upper gastrointestinal tract. Endosc Int Open.

[13] Loske G, Liedke M, Schlöricke E, Herrmann T, Rucktaeschel F (2017). Endoscopic negative-pressure therapy for duodenal leakage using new open-pore film and polyurethane foam drains with the pull-through technique. Endoscopy.

[14] Krajinovic K, Reimer S, Kudlich T, Germer CT, Wiegering A (2016). “Rendezvous technique” for intraluminal vacuum therapy of anastomotic leakage of the jejunum. Surg Case Rep.

[15] Loske G, Schorsch T, Rucktaeschel F, Schulze W, Riefel B, van Ackeren V, Müller CT (2018). Open-pore Film Drainage (OFD) – A new multipurpose tool for endoscopic negative pressure therapy (ENPT). Endosc Int Open.

[16] Wallstabe I, Tiedemann A, Schiefke I (2012). Endoscopic vacuum-assisted therapy of infected pancreatic pseudocyst using a coated sponge. Endoscopy.

[17] Loske G, Schorsch T, Gobrecht O, Martens E, Rucktäschel F (2016). Transgastric endoscopic vacuum therapy with a new open-pore film drainage device in a case of infective pancreatic necrosis. Endoscopy.

[18] Loske G, Rucktäschel F, Schorsch T, van Ackeren V, Stark B, Müller CT (2015). Successful endoscopic vacuum therapy with new open-pore film drainage in a case of iatrogenic duodenal perforation during ERCP. Endoscopy.

[19] Loske G, Schorsch T, van Ackeren V, Schulze W, Müller CT (2015). Endoscopic vacuum therapy in Boerhaave’s syndrome with open-pore polyurethane foam and a new open-pore film drainage. Endoscopy.

[20] Loske G, Schorsch T, Kiesow RU, Müller CT (2017). First report of urinary endoscopic vacuum therapy : For large bladder defect after abdomino-perineal excision of the rectum. Video paper. Chirurg.

[21] Loske G, Schorsch T, Müller CT (2017). Prevention of reflux after esophagectomy with endoscopic negative pressure therapy using a new double-lumen open-pore film drainage with an intestinal feeding tube. Endoscopy.

[22] Loske G, van Ackeren V, Denkhaus H, Müller C (2007). Vacuumschwammtherapie einer Ösophagusanastomoseninsuffizienz.

[23] Kirschner M (1926). Die Behandlung der akuten eitrigen freien Bauchfellentzündung. Langenbecks Arch Chir.

[24] Neumann PA, Mennigen R, Palmes D, Senninger N, Vowinkel T, Laukoetter MG (2017). Pre-emptive endoscopic vacuum therapy for treatment of anastomotic ischemia after esophageal resections. Endoscopy.

[25] Loske G, Lang U, Schorsch T, Müller CT (2015). Komplexe Vakuumtherapie einer abszedierenden Magenperforation. Chirurg.

